# The influence between gestational age and postural control, a systematic review

**DOI:** 10.3389/fped.2022.883218

**Published:** 2022-11-15

**Authors:** Isabel Tuñón-Domínguez, Alicia Cuesta-Gómez, Francisco Molina-Rueda, Raúl Juárez-Vela, Regina Ruiz de Viñaspre-Hernández, Iván Santolalla-Arnedo, Juan Luis Sánchez-González, María Carratalá-Tejada

**Affiliations:** ^1^ Hospital Beata María Ana, Madrid, Spain; ^2^ Motion Analysis, Ergonomics, Biomechanics and Motor Control Laboratory (LAMBECOM), Department of Physical Therapy, Occupational Therapy, Rehabilitation and Physical Medicine, Faculty of Health Sciences, Rey Juan Carlos University, Madrid, Spain; ^3^ Department of Nursing, University of La Rioja, Research Group GRUPAC, Logroño, La Rioja, Spain; ^4^ Department of Nursery and Physiotherapy, Faculty of Nursery and Physiotherapy, University of Salamanca, Salamanca, Spain

**Keywords:** postural control, gestational age, children, gestational age (GA), preterm

## Abstract

The central nervous system (CNS) of preterm infants might have some peculiarities which distinguish it from that of full term infants. The difficulties associated with prematurity are the main cause of deaths all over the world during the new-born period after community-acquired pneumonia, and the second cause of deaths worldwide in children under five years old. Early recognition of signs indicating fragile postural control in premature infants can support understanding and help prevent and early intervention on possible future neuromotor dysfunctions in these subjects. The purpose of this paper is to determine if there is a qualitatively different development of postural control in premature infants without neurological involvement and infants born at term. We conducted a systematic review of longitudinal and cross-sectional case-control studies published between 2010 and March 2020 on this topic. The evaluation of parameters related to postural control was also included. The methodological quality of the selected works was evaluated using the CASPe critical reading programme for cases and controls. PRISMA guidelines for systematic reviews were followed for prematurity and postural control. 16 articles were included. The total sample amounted to 3,460 participants, of which 1,860 in the preterm group, and 1,600 in the control group. All the studies found show a poorer postural control by the group of children born preterm compared to the group of children born at term and one study indicating more limited postural control with higher prematurity. Regarding the methodological quality according to CASPe, those studies exceeding half of the total score were considered of adequate quality.

## Introduction

The World Health Organization (WHO) defines as children born preterm before 37 weeks of pregnancy are completed (1). The difficulties associated with prematurity are the main cause of deaths all over the world during the new-born period (2). It is estimated that, in 2030, the mortality caused by premature labour difficulties will rise from 15% to 18; however, this will occur especially in low-incomes countries, where half of the children born at 32 weeks die because they do not receive proper care (3). On the other hand, there is a rise of preterm infant survival rate in developed countries, thanks to obstetrics and neonatology advances (4).

Furthermore, prematurity implies an important associated morbidity (5) and an inverse relationship with gestational age (5–8). A study carried out in 2019 concludes that the estimated probability of survival without disabilities up to 25 years old is 4.1% for children born at a gestational age of 22 weeks, 78.3% for those born at 28 weeks, and 97.2% for those born full term (9). It is estimated that approximately 8%–9% of children born between 22 and 32 weeks and 14% of those born between 22 and 25 weeks develop cerebral palsy (CP), this being one of the main associated pathologies (10).

Accordingly, the central nervous system (CNS) of preterm infants might have some peculiarities which distinguish it from that of full term infants. One of these peculiarities could be alterations in postural control.

Having knowledge of postural control in preterm infants can support the understanding of motor competence, thus helping the prevention of and early intervention on future neuromotor dysfunctions. To begin with, it should be understood that postural control has been defined as the act of maintaining, achieving, or restoring a state of balance during any posture or activity. Postural control strategies can be predictive or reactive, and can involve a response of fixed support or change of support (11, 12). Maintaining postural control requires the integration of the information provided by the vestibular, somatosensory, and visual systems (13, 14), in addition to the integrity of the cerebellum as the coordinator of the three systems. However, postural stability does not require only sensory integration, as adequate motor response is also necessary to maintain the effect of the centre of gravity inside the support surface (15). It is important to note that, postural control is primarily prospective (as opposed to reactive) and used to engage with the environment and to support action systems (manipulation, attention, locomotion, orienting); thus, intervention should focus on infant-directed action so that prospective control is inherent in the therapeutic plan. Postural control is the background of all other action systems and thus should be a primary focus as functional skills are changing over time (16).

Considering that a preterm newborn may present higher risk factors, we need to identify how motor development and the trajectory of postural acquisitions differ between preterm and full term infants, in order to provide more specific action protocols and procedure strategies to this vulnerable group. Our aim was to determine the effect of prematurity on the development of postural control during the postnatal/infancy stage in children born before 37 weeks and without disease or neurological sequelae after birth, as compared to healthy children born at term.

## Methods

We performed a Systematic Review of longitudinal and cross-sectional case-control studies included in the literature review, published from the year 2010 to March 2020, which confirm a possible relationship between prematurity and postural control. The research question was established according to the “population, intervention, comparison and outcome” model (PICO), where the inclusion criteria are in relation to the selection of studies. Regarding population, the participants of this review were limited to underage patients without diseases and neurological sequelae, divided into two groups according to gestational age; those children born preterm (<37 gestational weeks) made up the preterm group (PG), while those born full term were referred to the control group (CG). The intervention consisted in evaluating specific values of postural control, in order to make a comparison between the results obtained by the preterm group and those obtained by the control group. The measurement of the results included the evaluation of values related with postural control, such as control of the segmental trunk, movement quality in different positions, balance, manual dexterity, etc. Several limits were established concerning the language of the articles; English and Spanish were selected. As for dates of publication, all the articles from January 2010 to March 2020 were included. The following databases were checked: Scopus, Web of Science, CINHAL, Medline Complete, Science Direct, and PubMed. As to the search terms, the ones used, including the Medical Subject Heading (MESH), were: “premature infant”, “premature birth”, “preterm infants”, “full term infants”, “postural balance”, “balance” and “postural control”; all of them combined with the Boolean operators AND and OR. All related articles that used analogous terms that meant prematurity/preterm were considered.

The article selection process included some steps. The first step was to review titles and abstracts and exclude those not relevant to this study; duplicates were excluded next. As for the second step, complete texts were downloaded for review; only those complying with the inclusion criteria and answering the research question were selected. The third step consisted of a manual search to obtain references that might have not appeared during the first step.

All the potentially available articles were examined by two assessors who evaluated the selection independently, by analysing the full texts based on those which kept to the inclusion criteria with the aim of deciding their relevance to the review. The guidelines of the PRISMA statement were followed to improve the quality of this systematic review (17).

The methodological quality of the selected studies was evaluated according to the critical reading programme CASPe for case-control studies, which presents a total of 11 items. The first two are elimination questions, where both answers must be positive to be able to continue. Items 1–5 verify if the results of the study are valid, whereas items 6–9 show which results are obtained, while items 9–11 indicate if the acquired results are applicable. The answers to these items may be “yes”, “I don’t know” or “no”. One point is added for every item answered with a “yes”; “I don’t know” or “no” answers are not scored. Consequently, the highest score for an article cannot be higher than 11 points, and the minimum score may be 0 points. Studies with a higher score are considered to have a higher methodological quality than those with a lower score.

## Results

### Description of the studies

**Figure 1:**
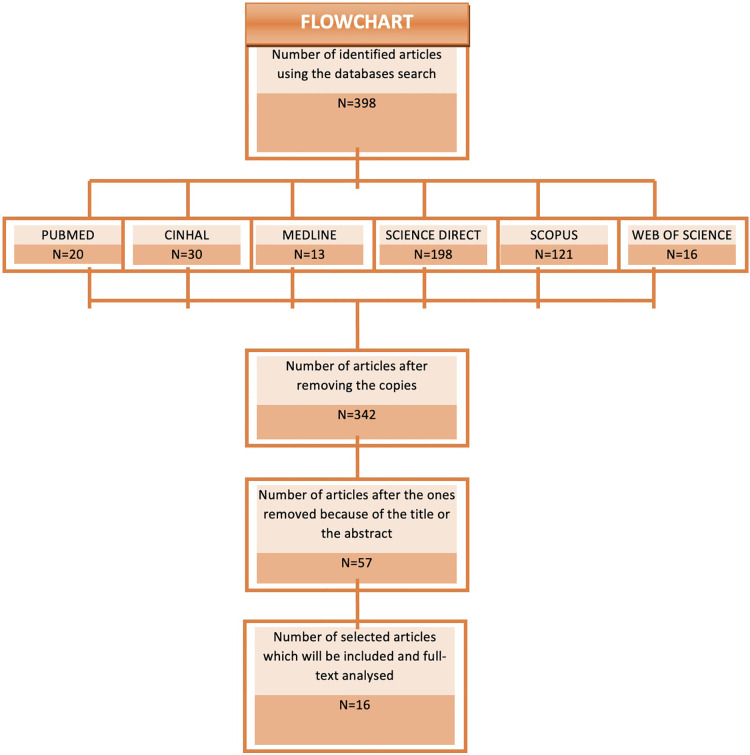
Flowchart.

Searching the different databases yielded 398 studies. After taking out the duplicates, and those which, based on their title or abstract, were not considered adequate to the inclusion criteria, we were left with a total of 57 studies. After an in-depth analysis, we finally included a total of 16 articles (18–33) in the study (Figure 1, Table 1).

**Table 1:** Results.

Author	Type of study	Participants	Evaluation tools and parameters	Assessment age	Main results
Pin et al. 2019 (18)	Prospective longitudinal case-control study.	PG (*n* = 31) AGA: 27.2 weeks ABW: 955.8 g Age: 4–12 months Sex: 15M/16F CG (*n* = 30) AGA: 38.9 weeks ABW: 3,007.4 g Age: 4–12 months Sex: 17M/13F	AIMS It evaluates the quality of movement **(alignment, postural control, load, balance, and antigravity movements)** in different postures (SP, PP, S, ST). SATCo It evaluates **the segmental control** (cervical, thoracic, and lumbar) **of the trunk and total control of the head and trunk**, considering the static, active and reactive control.	All infants were evaluated at 4, 8 and 12 months of age.	The PG has a significantly lower level of trunk control than the CG in the three evaluations carried out; the PG taking longer to develop the same level of trunk control. Statistically significant worse results are found in posture SP and ST at 4 months and in the total score at 12 months of age for PG. A significant correlation is shown between segmental trunk control and posture in SP, PP, S and ST at 8 and 12 months, but not at 4 months (considering the results of all the children as a single group).
Righetto et al. 2019 (19)	Cross-sectional case-control study.	PG (*n* = 26) AGA: 33.86 weeks ABW: 2,403 g Age: 6–7 months Sex: 19M/7F Apgar score: 8.33 (± 1.57) first minute Apgar score: 9.20 (± 1.47) fifth minute CG (*n* = 26) AGA: 39.53 weeks ABW: 3,305 g Age: 6–7 months Sex: 17M/9F Apgar score: 9 (± 0.78) first minute Apgar score: 9.8 (± 0.44) fifth minute	AIMS It evaluates the quality of movement **(alignment, postural control, load, balance, and antigravity movements**) in different postures (SP, PP, S, ST). SATCo It evaluates **the segmental control** (cervical, thoracic, and lumbar) **of the trunk and total control of the head and trunk**, considering the static, active and reactive control.	All infants were evaluated at 6.7 months of age.	The PG has a significantly lower level of trunk control and poorer performance in the PP, SP and S posture compared to the CG. Significant correlation between segmental trunk control and posture in SP, S, and AIMS total score for the CG and in all postures for the PG.
Valentini et al. 2019 (20)	Prospective longitudinal case-control study.	PG (*n* = 1,361) AGA: 22.07 weeks ABW: 1,840.64 g Age: 0–12 months Apgar score: 8.31 (±3.19) fifth minute CG (*n* = 1,218) AGA: 39.27 weeks ABW: 3,319.80 g Age: 0–12 months Apgar score: 8.82 (± 1.28) fifth minute	AIMS It evaluates the quality of movement **(alignment, postural control, load, balance, and antigravity movements**) in different postures (SP, PP, S, ST).	All infants were evaluated monthly for 12 months.	During the first trimester of life, the PG shows significantly higher results in the postures PP, SP, S and ST, (especially in the last two) compared to the CG. In the following months the PG shows a lesser variety of motor acquisitions and lower motor performance scores, specifically in SP at 9 months, S at 2 to 4 months, and PP and ST at 9 and 10 months.
Pin et al. 2018 (21)	Prospective longitudinal case-control study.	PG (*n* = 20) AGA: 27.2 weeks ABW: 989.6 g Age: 4–9 months Sex: 8M/12F Apgar score from 7 to 10 in the first and 15 min CG (*n* = 20) AGA: 38.7 weeks ABW: 3,019.9 g Age: 4–9 months Sex: 12M/8F Apgar score from 7 to 10 in the first and 15 min	SATCo It evaluates **the segmental control** (cervical, thoracic, and lumbar) **of the trunk and total control of the head and trunk**, considering the static, active and reactive control.	All infants were evaluated monthly from 4 to 9 months of age.	No significant differences were found between PG and CG in the trunk segmental control, except for the reactive trunk control at 8 months with lower scores for the PG.
Sato et al. 2018 (22)	Prospective longitudinal case-control study.	PG (*n* = 20) GA: 32–37 weeks Average age: 6 months CG (*n* = 36) GA: 37–41 weeks Average age: 5.8 months	SATCo It evaluates **the segmental control** (cervical, thoracic, and lumbar) **of the trunk and total control of the head and trunk**, considering the static, active and reactive control.	The infants were evaluated once a month until 8 months.	Both groups show segmental trunk control acquired in the cephalocaudal order, the PG shows worse results for trunk control and is slower in its acquisition. Regarding the influence of the sitting position of the ring and the 90° of flexion within reach, we found that there were no significant differences for most of the spatial and temporal variables between both groups, showing the PG makes more attempts and has a less straight trajectory than the CG.
Bucci et al. 2017 (23)	Prospective longitudinal case-control study.	PG (*n* = 29) AGA: 26.3 weeks ABW: 840 g Average age: 5.38 years Sex: 16M/13F CG (*n* = 29) AGA: 39.2 weeks ABW: 3,700 g Average age: 5.38 years Sex: 15M/14F	Stable and unstable platform (Framiral®) It evaluates **visuospatial skills:** eyes open, eyes closed, and eyes open with disturbed vision; and **static and dynamic balance.** The spatial analysis of postural performance is considered: **surface of the centre of pressure (CoP) and the mean velocity of the CoP;** and temporal analysis of postural performance: **cancellation time (CT) and spectral power index.**	All infants were evaluated between 5 and 6 years of age.	The surface and the mean velocity of the centre of pressure are significantly higher in the PG compared to the CG, especially in an unstable posture condition and with eyes closed. The spectral power index was significantly higher for PG than for CG for all frequencies, especially with disturbed vision and high frequency under unstable conditions. No significant differences were found for the cancellation time in the anteroposterior direction between the two groups.
Dziuba et al. 2017 (24)	Cross-sectional case-control study.	PG (*n* = 59) GA: 24–35 weeks Age: 6–7 years Sex: 28M/31F CG (*n* = 61) GA: >37 weeks Age: 6–7 years Sex: 33M/28F	*The one-leg jumping test* *The one-leg-open-eyed test* *Closed-eyed standing tests:* It evaluates **dynamic and static balance with both eyes open and eyes closed and jump with single leg and both legs.**	All infants were evaluated between 6 and 7 years of age.	There are no statistically significant differences between the two groups in terms of dynamic balance, static balance, or total balance control. Comparison of the tests performed on the right and left lower limb in the CG shows statistically significant differences for static balance and total balance control, but not for dynamic balance, and no significant difference for the PG.
Rodríguez et al. 2016 (25)	Cross-sectional case-control study.	PG (*n* = 59) GA: ≤37 weeks BW: ≤1,500 g Age: 7–10 years Sex: 26M/33F Apgar score: 6.9 (± 2.3) first minute Apgar score: 8.1 (± 2.4) fifth minute CG (*n* = 30) GA: ≥37 weeks BW: ≥2,500 g Age: 7–10 years Apgar score: 9 (±0.4) first minute Apgar score: 9.94 (±0.2) fifth minute	MABC-2 It evaluates **manual dexterity, aiming and ball catching, and balance.** Stabilometry: *Metitur Good Balance®* It evaluates s**tatic balance with eyes open and closed, feet together and apart, and with and without foam cushioning on the support surface.**	All infants were evaluated between 7 and 10 years of age.	The PG obtained worse statistically significant total scores globally in manual dexterity and balance on the MABC-2, but not for marksmanship and capture. The balance was similar through stabilometry between both groups, except for the closed eyes and non-foam support surface, with worse results for the PG.
Silva et al. 2016 (26)	Cross-sectional case-control study.	PG (*n* = 10) GA: 28–36 weeks Age: 3 years Sex: 4M/6F CG (*n* = 10) GA: >37 weeks Age: 3 years Sex 4M/6F	DMS: It evaluates **gross and fine motor skills, balance, body schematics, spatial awareness, and temporal awareness.**	All infants were evaluated at 3 months of age.	Significant differences are shown in fine motor skills, and in spatial and temporal awareness, with the results of the group of premature children being poorer. No differences are found in the values of the body scheme and balance.
Pertersen et al. 2015 (27)	Cross-sectional case-control study.	PG (*n* = 21) GA: <34 weeks BW: <2,500 g Average age: 17.2 years Sex: 3M/18F CG (*n* = 21) GA: >37 weeks BW: >2,500 g Average age: 17 years	Posturography: It evaluates **active, reactive, and anticipatory balance with eyes open and closed.**	All infants were evaluated at 17 years of age.	Active and reactive postural stability in the anteroposterior direction shows significantly worse results in the PG with both eyes closed and eyes open. Postural stability in the lateral direction shows significantly worse PG results, although here only during standing tests with eyes open.
Bucci et al. 2015 (28)	Cross-sectional case-control study.	PG (*n* = 22) AGA: 26.4 weeks ABW: 863.6 g Average age: 16.5 months Sex: 16M/6F CG (*n* = 22) AGA: 39.1 weeks ABW: 3,670 g Average age: 13.2 months Sex: 15M/7F	Techno Concept platform: It evaluates the postural control of MM SS considering mean of **the CoP surface, length of the CoP in the mid-lateral axis and mean velocity of the CoP.**	All infants were evaluated between 13 and 16 months of age.	Postural performance shows poorer results in PG compared to term children in terms of surface area, length in the mid-lateral direction, and mean velocity of the centre of pressure (CoP).
Cabral et al. 2015 (29)	Cross-sectional case-control study.	PG (*n* = 15) AGA: 31.3 weeks ABW: 1,506 g Average age: 22.2 weeks Sex: 7M/8F Apgar score: 4.9 (± 1.6) first minute Apgar score: 8.7 (± 1.0) fifth minute CG (*n* = 15) AGA: 39,.2 weeks ABW: 3,047.3 g Average age: 19.8 weeks Sex: 8M/7F Apgar score: 6.8 (± 3.5) first minute Apgar score: 7.7 (± 4.0) fifth minute	TSFI It evaluates the behaviour of sensory integration: **reactivity to tactile pressure, adaptive motor response, visuo-tactile integration, oculomotor control and reactivity to vestibular stimulation.** AIMS It evaluates the quality of movement **(alignment, postural control, load, balance, and antigravity movements**) in different postures (SP, PP, S, ST).	All infants were evaluated between 19 and 22 weeks of age.	In the comparison between the groups with respect to the motor development evaluated by means of the AIMS scale, there are no significant differences in the total score and in the PP and SP postures, although differences are shown in sensory processing. Regarding the correlation between intragroup motor development and sensory processing, there was no significant association between the AIMS and TSFI total scores.
Eshaghi et al. 2015 (30)	Cross-sectional case-control study.	PG (*n* = 31) GA: 28–37 weeks Age: 5–6 years Sex: 18M/13F CG (*n* = 20) GA: >37 weeks Age: 5–6 years Sex: 9M/11F	BOT-2 It evaluates **static and dynamic balance with eyes open and closed.**	All infants were evaluated between 5 and 6 years of age.	The mean scores on the four BOT-2 static balance subscales were significantly lower in PG compared to CG.
Lorefice et al. 2014 (31)	Cross-sectional case-control study.	PG (*n* = 90) AGA: 27.3 weeks ABW: 1,022 g Age: 4 years Sex: 45M/45F CG (*n* = 40) AGA: 39.4 weeks ABW: 3,507 g Age: 4 years Sex: 17M/17F	Posturography: (*Wii Balance Board)* It evaluates **static and dynamic balance with eyes open and closed, adding dual cognitive task or foam mat on the surface, monopodal support with both legs, vertical jump with double support or monopodal support.**	All infants were evaluated at 4 years of age.	PG is shown to have impaired static and dynamic balance and reduced flight times in double-stance vertical jump and left-leg stance jump compared to CG. When the results are adjusted for body weight, the only group differences in postural control are found in the dual cognitive task and in the flight times of the vertical jump with double support and left leg support.
Dusing et al. 2014 (32)	Retrospective longitudinal case-control study.	PG (*n* = 18) AGM: 28.3 weeks PNM: 1,178 g Age: 0–6 months Sex: 8M/10F GC (*n* = 22) GM: 39 weeks ABW: 3,311 g Age: 0–6 months Sex: 10M/12F	CONFORMat® It evaluates the **postural control of the head in the midline and the reach, considering the magnitude of the variability of the CoP shift and the complexity of the CoP time series.**	The evaluation is done twice a month until 3 months of age, and once a month from 3 to 6 months.	The PG uses more repetitive and less adaptive postural control strategies compared to the CG. Both groups changed their postural complexity used during the development of head control and reach.
Wang et al. 2010 (33)	Retrospective longitudinal case-control study.	PG (*n* = 93) AGM: 29.14 weeks ABW: 1,136.03 g Age: 6 and 12 months Apgar score: 5-min CG (*n*=) no information. Age: 6 and 12 months	NMI AIMS AIMS It evaluates the quality of movement **(alignment, postural control, load, balance, and antigravity movements**) in different postures (SP, PP, S, ST).	The evaluation is carried out at 6 and 12 months.	The PG has statistically significant worse results than the CG at both 6 and 12 months. The GP has proportionally more delay in the development of postural control at 6 months than at 12 months.

PG, preterm group; GA, gestational age; AGA, average gestational age; BW, birth weight; ABW, average birth weight; CG, control group; M, male; F, female; SP, supine position; PP, prone position; S, sitting; ST, standing; AIMS, the Alberta infant scale; SATCo, the segmental assessment of trunk control; MABC-2, the movement assessment battery for children-second edition; DMS, motor development scale-Francisco Rosa Nieto; TSFI, the test of security functions; BOT-2, Bruininks–Oseretsky test of motor proficiency-second edition; TIMP, the test of infant motor performance; Bayley, Bayley scale of infant and Toddler development; NMI, neonatal medical index.

The studies were of an observational nature, case-control, which referred to preterm and full-term infants, respectively.

Altogether, there was a sample of 3,460 participants, 1,860 in the children born preterm group and 1,600 in the control group, referring to those children born at term, although it should be considered that one of the studies did not provide data about the number of participants in the control group (33). All the studies included underage participants, who were classified according to gestational age, regardless of birth weight and sex. Out of the 16 studies, eight provided the average gestational age per group (19–21, 23, 28, 29, 31, 32); seven provided the intervals of gestational age which corresponded to each group (18, 22, 24–27, 30), and one provided the data about the average gestational age of the preterm group, even though it did not provide data referring to the control group (33). The birth weight and sex of both groups was specified in 10 studies. All the studies specified the intervention age, except for one (33), which only made reference to the age of the preterm group. Considering the last condition, it may be observed that seven studies were carried out during the first year of life of the participants (18–22, 29, 32, 33), six were carried out from 3 to 10 years old (23, 24, 26, 30, 31), two were developed at the age of 13–17 years old (25, 27, 28) and one was carried out during the first year of life of the preterm infants group, but here the age of the control group was not specified (33). Out of the 16 studies included, six followed the evolution of the participants during a determined period of time; four were of a prospective nature (18, 20–22), and two of a retrospective nature (32, 33); the rest were cross-sectional studies.

Regarding the evaluation tools, six studies used posturography; four used it exclusively (23, 27, 28, 31); one used it together with the Movement Assessment Battery for Children-second edition (MABC-2) (25); the last one used it together with the Test of Infant Motor Performance (TIMP) and Bayley Scale of Infant and Toddler Development (Bayley) (32). The Alberta Infant Motor Scale (AIMS) was used by five studies; one used it exclusively (20); another used it together with the Test of Security Functions (TSFI) (29); a third study used it together with the Neonatal Medical Index (NMI) (33); and the two remaining studies used it together with the Segmental Assessment of Trunk Control (SATCo) (18, 28). Additionally, two studies carried out the evaluation of SATCo exclusively (21, 22). As for the evaluation tools, the Motor Development Scale-Francisco Rosa Nieto (MDS) (26), Bruininks-Oseretsky Test of Motor Proficiency-second edition (BOT-2) (30), the one-leg jumping test, the one-led-open-eyed test, and the closed-eyed standing test (24) were also used.

### Synthesis of the main results

The results obtained from the statistical analysis of all the studies were considered as significant when *p* < 0.05. Segmental trunk control was evaluated using SATCo, in Pin et al. (18) and Righetto et al. (19), which found worse results during the first year of life for the preterm group than for the full-term group. Pin et al. (21) found worse results for the preterm group only in one item of SATCo (in reactive trunk control), in the articles in which this measure was used, the participants analyzed were in the first year of life. Pin et al. (18) and Sato et al. (22) confirmed that the learning processes in the preterm group were slower. Pin et al. (18) and Righetto et al. (19) checked for correlations between trunk control and gross motor skills during the first year of life. The first study found a significant correlation between trunk control and movement quality in every position at 8 and 12 months old, but not at 4 months old, whereas the second study found a significant correlation between trunk control and the supine and sitting positions, and in the total score of AIMS for the control group, and in every position for the preterm group.

As for movement quality in different positions, which was evaluated by means of AIMS, in the articles in which this measure was used, the participants analyzed were in the first year of life. Pin et al. (18) found lower results for the preterm group in the total score at 12 months old; Wang et al. (33) did as well, at 6 and 12 months old. Significant differences which means worse results for the preterm group were also found: by Pin et al. (18) in sitting and bipedalism at 4 months old; by Righetto et al. (19), in the supine, prone and sitting position at 6–7 months old; by Valentini et al. (20) in the supine position at 9 months old, sitting position at 2 to 4 months old and in bipedalism and the prone position at 9 and 10 months old; and by Cabral et al. (29) in the supine and prone position at 5 months old. However, Valentini et al. (20) found that, during the first term of life, the preterm group obtained higher results in every position, but during the next months the control group showed a wider variety of motor acquisitions and higher scores in motor performance.

On the other hand, balance was evaluated in seven studies (23–28, 30, 31), the children included in these studies had ages from three years. Dziuba et al. (24) and Silva et al. (26) did not find significant differences, and Rodríguez et al. (25) did not find significant results for stabilometry except for the closed-eyes condition and over a viscoelastic foam surface (which were worse for the preterm group), but they did find significant differences for MABC-2. The study by Bucci et al. (23), which considered the surface and the average speed of the centre of pressure (CoP), obtained worse result for the preterm infants group; whereas Petersen et al. (27) showed by means of posturography worse results in the active and reactive postural stability of the preterm group with open and closed eyes in an anteroposterior direction and with open eyes in a lateral direction. Eshagui et al. (30) found that the average scores in the four static balance subscales of BOT-2 were significantly lower in the preterm infants group. Lorefice et al. (31) found that the preterm group had a damaged static and dynamic balance and limited jumping times in vertical double-support jumping and left-leg-support jumping in comparison with the control group.

Lastly, manual dexterity or range were evaluated in four studies (22, 25, 28, 32). Sata et al. (22) and Dursing et al. (32) studied this factor in children in their first year of life, while the children included in the studies by Rodriguez et al. (25) and Bucci et al. (28) comprised ages between 13 and 17 years.

Sata et al. (22) observed more range attempts and a lower straight trajectory to the midfield line for the preterm group than for the control group. Dursing et al. (32) showed that the preterm infants group used more repetitive and less adaptive postural control strategies in comparison with the control group and that both groups changed their postural complexity during the development of the head and range control.

Rodríguez et al. (25) found worse total scores in the preterm infants group in manual dexterity, although not in aiming and ball-catching. Bucci et al. (28), referring to the postural performance of the upper member, showed worse results in the preterm group, regarding the surface area, the medial-lateral direction length, and the average speed of CoP.

### Methodological quality

The methodological quality of the studies included in an article was evaluated using the critical reading programme CASPe (Table 2), based on which a maximum score of 9/11 points was obtained in five studies (19, 20, 23, 30, 32), 8/11 points in six studies (18, 22, 25, 27, 28, 31), 7/11 point in four studies (21, 24, 29, 33), and 6/11 point in one study (26). Those with a higher score were considered to have better methodological quality.

**Table 2:** Quality assessment: case-control critical Reading programme CASPe.

	1	2	3	4	5	6	7	8	9	10	11	Total
Pin et al. 2019 (18)	Yes	Yes	Yes	Yes	Yes	No	Yes	Yes	Yes	No	Yes	9
Righetto et al. 2019 (19)	Yes	Yes	Yes	Yes	Yes	Yes	Yes	Yes	Yes	No	Yes	10
Valentini et al. 2019 (20)	Yes	Yes	Yes	Yes	Yes	Yes	Yes	Yes	Yes	No	Yes	10
Pin et al. 2018 (21)	Yes	Yes	Yes	No	Yes	No	Yes	Yes	Yes	No	Yes	8
Sato et al. 2018 (22)	Yes	Yes	Yes	Yes	Yes	No	Yes	Yes	Yes	No	Yes	9
Bucci et al. 2017 (23)	Yes	Yes	Yes	Yes	Yes	Yes	Yes	Yes	Yes	No	Yes	10
Dziuba et al. 2017 (24)	Yes	Yes	Yes	Yes	No	Yes	Yes	Yes	Yes	No	No	10
Rodríguez et al. 2016 (25)	Yes	Yes	Yes	¿	Yes	Yes	Yes	Yes	Yes	No	Yes	9
Silva et al. 2016 (26)	Yes	Yes	Yes	Yes	No	No	Yes	Yes	Yes	No	No	10
Pertersen et al. 2015 (27)	Yes	Yes	Yes	Yes	Yes	No	Yes	Yes	Yes	No	Yes	9
Bucci et al. 2015 (28)	Yes	Yes	Yes	Yes	Yes	No	Yes	Yes	Yes	No	Yes	9
Cabral et al. 2015 (29)	Yes	Yes	Yes	No	Yes	No	Yes	Yes	Yes	No	Yes	8
Eshaghi et al. 2015 (30)	Yes	Yes	Yes	Yes	Yes	Yes	Yes	Yes	Yes	No	Yes	10
Lorefice et al. 2014 (31)	Yes	Yes	Yes	Yes	Yes	No	Yes	Yes	Yes	No	Yes	9
Dusing et al. 2014 (32)	Yes	Yes	Yes	Yes	Yes	Yes	Yes	Yes	Yes	No	Yes	10
Wang et al. 2010 (33)	Yes	Yes	Yes	?	Yes	No	Yes	Yes	Yes	No	Yes	8

Yes = 1 point.

Unknown (?) = 0 points.

No = 0 points.

Questions of Case-control critical reading programme CASPe: 1. Did the study address a clearly focused issue?; 2. Did the authors use an appropriate method to answer their question?; 3. Were the cases recruited in an acceptable way?; 4. Were the controls selected in an acceptable way?; 5. Was the exposure accurately measured to minimise bias?; 6. Aside from the experimental intervention, were the groups treated equally?; 7. How large was the treatment effect?; 8. How precise was the estimate of the treatment effect?; 9. Do you believe the results?; 10. Can the results be applied to the local population?; 11. Do the results of this study fit with other available evidence?

## Discussion

The aim of this study was to carry out a literature review, with the goal of understanding whether the development of postural control of preterm infants without neurological sequelae is different from that of full-term infants, and whether there exists any relationship between prematurity and postural control. All the studies we found compared the postural control of preterm and full-term infants; in addition, one of the studies also checked whether more prematurely born infants have worse postural control than those less prematurely born. The results obtained by the studies showed that the preterm group has worse postural control than the control group.

The findings also showed worse trunk control by the preterm infants group, with slower learning processes. All the studies involving this value considered the improved age, meaning if the baby had been born with 40 weeks of gestation, recommended in order to compensate for biological immaturity until the child is able to walk without help (34), which, added to the equality of the patients' functional status at the beginning of the treatment, confirms the absence of confusion bias. Segmental trunk control was evaluated with SATCo during the first year of life, which is an authentication tool for three different terms in a sitting position: continuity in a neutral vertical position without movement (static control), continuity in a neutral vertical position during voluntary movements of the head or range (active control), and recovery of a neutral position after a balance disturbance due to a push (reactive control). Furthermore, this scale is considered as highly reliable among assessors (ICC ≥ 0.8) (35). The studies carried out by Pin et al. (18) and Righetto et al. (19) showed the same results even though the gestational age in the second study was higher than in the first. However, Pin et al. (18) and Pin et al. (21) had different results in the evaluation of segmental trunk control at 4 and 8 months old. The first study showed worse significant results for the preterm infants group in static, active and reactive control in both evaluations, while the second study only showed worse results in reactive trunk control at 8 months old; although both studies included extremely preterm infants in the preterm group, and used a similar sample size and the same evaluation tool. Pin et al. (18) and Righetto et al. (19) checked for statistically significant correlations between trunk control and gross motor skills during the first year of life, using SATCo and AIMS, and showed coefficients varying between 0.86 and 0.88 (35). However, the first study verified this correlation independently of the gestational age, whereas the second study distinguished the control group from the preterm group, which is the reason why the difference between the findings obtained in both studies can be justified.

As regards movement quality in different positions, worse results were obtained for the preterm infants group. All the evaluations were done using AIMS, a scale authenticated for preterm infants considered to be highly reliable by assessors (ICC ≥ 0.99) (36). Therefore, the implementation of this scale is appropriate, and it should be considered as a positive aspect that every study which evaluated postural control considered the improved age. The evaluations carried out using this scale were in children who were in their first year of life. Valentini et al. (20) found that during the first term of life, the preterm group obtained higher significant results in every position, whereas, during the next months, the preterm group had a smaller variety of motor acquisitions and lower scores in motor performance in comparison with the control group. According to the authors, a possible explanation for the preterm group showing better postural control results during the first term of life may be based on the fact that preterm new-borns, who do have difficulties integrating and modulating stimuli at birth, develop strategies to deal with their organic disadvantages and thus adapt to the environment through behavioural organization and intense motor maturity during the first months. However, this finding did not coincide with the results obtained in any other study, possibly because this study used a far wider sample than all the other studies. Contrary to the above, results revealing worse postural control for the preterm group during the next months of life were found by Wang et al. (33) at 6 and 12 months old, and Pin et al. (18), only at 12 months old, although no significant difference was found at 4 and 8 months old. The rest of the studies did not show significant results for the total score but only for specific positions. Moreover, the study carried out by Wang et al. (33) indicated that the preterm group had proportionally more deficiency in postural control at 6 than at 12 months old, which should be verified by future research.

With regard to balance, the majority of studies obtained unfavourable results for the preterm group. Rodríguez et al. (25), in their patients between 13 and 17 years old, found significant differences for MABC-2 between the two groups but did not find different statistically significant results for stabilometry (except for the closed-eyes condition and the support surface without foam), which is considered as the gold standard for this value in postural control evaluation (37). The disagreement observed between the two balance evaluation tools may be due to the kind of movement analysed, because stabilometry specifically evaluates postural control and adaptive response to various changes controlled in the sensory input, whereas MABC-2 includes exercises which involve not only postural control, but also other variables such as agility. On the other hand, the study carried out by Bucci et al. (23), which also used posturography in children with an average age of 5.38 years, found unfavourable results for the preterm group especially under the closed-eyes condition, although in this case the differences obtained in the unstable condition also stood out, as opposed to the results obtained by the previous study for the support surface without foam condition. These differences might have occurred because the two studies used different samples as far as age and type of platform used. The research carried out by Petersen et al. (27), that included children with an average age of 17.2 years, also used posturography as the evaluation tool; as with the previous studies, unfavourable significant results were obtained for the preterm group in the closed-eyes condition, but also in the open-eyes condition. It should be considered that this study used an older age sample than the previous two as well as a different kind of platform. In the case of the studies carried out by Eshaghi et al. (30) and Lorefice et al. (31), the preterm group, with children from three years, was found to have worse balance than the control group, even though these two studies used different evaluation tools. Lorefice et al. (31) used posturography, whereas Eshaghi et al. (30) used BOT-2, a scale which should be used starting at 4 and a half years and has good reliability (test-retest; ICC = 0.56) and moderate intern consistency (ICC = 0.67). In contrast with these results, the findings obtained by Dziuba et al. (24) and Silva et al. (26), that included children from three year, did not show significant differences between the preterm and full-term groups, although using different sample ages and evaluation tools. Furthermore, it should be considered that the Silva et al. (26) study carried out the evaluation of the preterm group under different conditions than for the control group; the preterm infants were evaluated at home in an environment where the evidence may have been gathered without outside influences, whereas the full-term infants were evaluated at school.

Finally, regarding manual dexterity or range, in total, the preterm group showed worse results than the control group, although with differing samples, average gestation periods, and evaluation tools.

In addition, the degree of prematurity was only considered by Eshaghi et al. (30), who distinguished between extremely preterm infants (average gestational age of 30.60 weeks) and very preterm infants (average gestational age of 34.9 weeks). The age of the sample evaluation comprised 5 and 6 years old, which means the application of BOT-2 as an evaluation tool can be considered adequate although not the gold standard, since this scale should be applied by the age of 4 and a half. However, only 10 extremely preterm infants were evaluated opposite to 21 very preterm infants. Statistically significant results were obtained between both groups with respect to scores for exercises consisting of standing on a straight line with eyes closed for 10 s, and on one leg on a balance beam with eyes closed. This demonstrates that degree of prematurity may be related to postural control. Further studies with a wider sample are needed in this case.

Moreover, it is important to keep in mind that a correct development of postural control depends on a great deal on the vestibulospinal component of vestibular function in early childhood. Vestibulospinal input is important for muscle power regulation, which, in turn, influences postural control. Even though, de Graaf et al. (38), in their study they focused on vestibular function during the first year of life in 67 infants with a very short gestational age (25–27 weeks) At the age of 3 months, 20 infants performed optimally on all items testing vestibular function, increasing to 40 at 6 months and 48 at 12 months. This significant improvement (also seen in muscle power regulation) was primarily caused by better head control (during the traction response and prone position), whereas less shoulder retraction and hyperextension were found in the sitting position. Vestibular function was significantly related to brain ultrasonography classification but not to gestational age, birthweight, the Neonatal Medical Index, or gender. It should be noted that, the developing nervous system has a great potential for plasticity. Functional and anatomic evidence demonstrates that spontaneous plasticity can be potentiated by activity and specific experimental manipulation. Particular attention should be paid to early detection from the clinical continuum of detection, diagnosis, prognosis, and intervention to improve developmental outcome. Therefore, one of the future lines of research that has not been included in this work would be the relationship between the vestibular system, the development of motor control and children born preterm.

One of the limitations of this bibliographic review is that only one of the articles described blinded methodology, as observational type studies do not normally carry out randomization processes and use limited size samples.

Moreover, only one study checked for the correlation between degree of prematurity and postural control, and a few studies measured long-term postural control, which allows to confirm the evolution of postural control differences through the years.

## Conclusions

In conclusion, all the studies that we found, demonstrated that the preterm infants group had worse postural control than the full-term infants group, with only one study indicating more limited postural control with higher prematurity. As for the methodological quality, all the studies exceeded a score of 7 according to the critical reading programme CASPe, which considers studies to have adequate methodological quality when they exceed half of the total score.

## Data Availability

The raw data supporting the conclusions of this article will be made available by the authors, without undue reservation.
